# Psychological interventions for the treatment of depression, anxiety, alcohol misuse or anger in armed forces veterans and their families: systematic review and meta-analysis protocol

**DOI:** 10.1186/s13643-017-0513-8

**Published:** 2017-06-15

**Authors:** Luke O’Shea, Ed Watkins, Paul Farrand

**Affiliations:** 1 Mood Disorders Centre, School of Psychology, College of Life and Environmental Sciences, University of Exeter, Exeter, UK; 2 Clinical Education Development and Research (CEDAR), Psychology: College of Life and Environmental Sciences, University of Exeter, Exeter, UK

**Keywords:** Veteran, Armed forces, Military, Psychological, Anger, Alcohol misuse, Depression, Anxiety, Systematic review, Military family

## Abstract

**Background:**

Evidence highlights a high prevalence of common mental health disorders in armed forces veterans and their families, with depression, anxiety, alcohol misuse and anger being more common than PTSD. This paper presents a protocol for a systematic review and meta-analysis to identify existing randomised controlled trial (RCT) research testing the effectiveness of psychological interventions for these difficulties in armed forces veterans and their family members.

**Methods:**

Electronic databases (CENTRAL, PsycInfo, MEDLINE, CINAHL, The Cochrane Register of Clinical Trials, EMBASE and ASSIA) will be searched to identify suitable studies for inclusion in the review supplemented by forward and backward reference checking, grey literature searches and contact with subject authors. Research including armed forces veterans and their family members will be included in the review with research including serving personnel or individuals under the age of 18 being excluded. Few RCTs examining the treatment of depression, anxiety, alcohol misuse or anger exist in armed forces veterans to date. The primary outcome will be symptomatic change following intervention for these difficulties. The secondary outcomes will include methodological aspects of interest such as discharge type and recruitment setting if data permits. In the event that the number of studies identified is too low to undertake a meta-analysis, a narrative review will be conducted. Quality assessment will be undertaken using the Cochrane Collaboration Tool and Cochran’s Q statistic calculated to test for heterogeneity as suggested by the Cochrane handbook.

**Discussion:**

The review will examine the findings of existing intervention research for depression, anxiety, alcohol misuse or anger in armed forces veterans and their families, along with any effect sizes that may exist.

**Systematic review registration:**

PROSPERO CRD42016036676

**Electronic supplementary material:**

The online version of this article (doi:10.1186/s13643-017-0513-8) contains supplementary material, which is available to authorized users.

## Background

The most common mental health disorders in the general population are depression, generalised anxiety disorder, social anxiety, obsessive compulsive disorder, panic and post-traumatic stress disorder (PTSD) [1]. Growing evidence exists that some of these disorders are also highly prevalent in armed forces veterans [2], with depression, anxiety and alcohol misuse highly prevalent, and more common than PTSD [3, 4]. Another common problem for armed forces veterans is anger that can inhibit psychosocial functioning and lead to loss of emotional support [5]. It is clear from the research to date that certain mental health difficulties prevalent in the general population are also prominent in armed forces veterans. Further investigation examining interventions targeting these difficulties is therefore warranted.

Despite the prevalence of these difficulties, many armed forces veterans do not seek mental health treatment [6]. One of the causes of this may be the anticipation of stigma, which remains a powerful barrier preventing members of the UK armed forces accessing help [7]. Not seeking treatment may exacerbate difficulties for some individuals, as those who are discharged from service with psychological problems are at high risk of experiencing ongoing ill health [3]. One group in which this may be particularly prominent is early service leavers (those who leave before the minimum term of their contract) who have poorer mental health and are at higher risk of developing mental health disorders than non-early service leavers [8]. No existing review takes the mental health of early service leavers into account; therefore, an integration of existing research addressing early service leavers or ways of altering interventions to promote participation and reduce barriers to treatment is required.

The high prevalence of common mental health difficulties is not only limited to armed forces veterans but is also experienced in their spouses and family members. This is due to the unique daily stressors they face, such as relocations and separations that affect all family members [9–11]. Given that research indicates a high prevalence of mental health difficulties in the family members of armed forces veterans, this review will focus on research addressing interventions in this population as well.

Whilst little is known about the mental health treatment of the family members of armed forces veterans, two systematic reviews exist that examine mental health treatments for armed forces veterans. The existing systematic reviews have found cognitive behavioural therapy (CBT) and psychosocial interventions effective for the treatment of mental health disorders in armed forces veterans. Kitchiner et al., [12] included 29 randomised controlled trials (RCTs) examining the efficacy of psychosocial interventions for anxiety and depressive disorders in armed forces veterans. They found telephone support effective for the treatment of depression and at risk drinking in two of the RCTs. Hundt et al., [13] included nine studies (five RCTs and four open trials) examining the effectiveness of CBT for depression in armed forces veterans. The open trials reported larger effect sizes, which may be due to them having fewer participants or arising as a consequence of confounding methodological factors, with the open trials of lower methodological quality than the RCTs. Furthermore, only two of the five RCTs found CBT more effective than wait-list and psychoeducational control conditions.

Despite these findings, the existing reviews are dated as they were conducted in 2012 and 2014. In addition to this, one review was limited to CBT for depression only, and neither review addressed anger or the mental health of the families of armed forces veterans. A broader, more up to date review of the topic area is therefore warranted.

In developing services and guiding future research, it is imperative to know the range of evidence-based treatments that are available and have been evaluated in armed forces veterans and their family members. In order to broaden the scope of the proposed review, a diverse list of search terms has been developed that will examine the treatment of depression, anxiety, alcohol misuse or anger in armed forces veterans and their families. This will facilitate a comprehensive review of research in the area of mental health treatments for depression, anxiety, alcohol misuse or anger in armed forces veterans, including early service leavers, and their families.

## Methods

Informed by PRISMA-P (Additional file 1) and the Cochrane handbook for systematic reviews [14, 15], this protocol was registered a priori with PROSPERO.

### Types of study design

The review will include studies with an RCT design, with both active and inactive control groups. This will allow causal determinants around the effectiveness of existing interventions to be reliably explored.

### Participants

Participants will include armed forces veterans and family members reporting symptoms of depression, anxiety, alcohol misuse or anger. There is a need to operationalise the term ‘veteran’ in this review as the term differs from country to country. For instance, the Canadian government considers a veteran to be ‘any former member of the Canadian armed forces released with an honourable discharge and who successfully completed basic training’ [16]. This is similar to the US definition of a ‘person who served in the active military, naval or air service, and was discharged or released therefrom under conditions other than dishonourable’ [17]. This review will use the more inclusive British definition of ‘Those who have served for at least one day in HM Armed Forces, whether as a regular or reservist’, which may lead to the inclusion of individuals who would not have been considered a veteran if an alternative definition were applied, and includes early service leavers [18].

### Intervention

Studies using psychological or psychosocial interventions for one of the target disorders/behaviours will be included in the review. A psychological intervention is a treatment that works to modify thoughts, feelings, behaviour or emotional state. “A ‘psychosocial intervention’ is a broad term used to describe different ways to support people to overcome challenges and maintain good mental health. Psychosocial interventions do not involve the use of medication” ([19], p2.).

Interventions may be delivered face-to-face as high-intensity psychological therapy or in a low-intensity format via the use of CBT self-help interventions with/without support provided face-to-face, over the telephone or in a written format or a combination of these support modalities. In order to reduce bias associated with short-term posttreatment effects, effect size will be calculated from data obtained both at immediate posttreatment up to 6 months follow-up.

Studies that report pharmaceutical interventions plus therapy intervention groups will be included in the review; however, those focusing solely on psychopharmacological interventions will be excluded.

### Comparators

Potential comparators could include treatment as usual, alternative treatment, watchful waiting, attention control or information provision groups.

### Outcomes

The primary outcome in this review will be symptomatic change in depression, anxiety, anger or alcohol misuse in armed forces veterans or their family members on standardised measures between intervention and control groups post intervention and up to 6 months post intervention. Symptomatic change may be from formal diagnosis or observer-rated or self-report measures such as PHQ-9, GAD-7, AUDIT and NAS-PI [20–23]. Outcomes post intervention up to and including 6 months post intervention will be extracted to enable a potential moderator analysis on length of follow-up. Secondary outcomes will include methodological aspects of interest such as comparator group or recruitment settings or follow-up outcomes which will allow the impact these factors may have in relation to primary outcomes to be explored. Effect sizes will be calculated using the Campbell Collaboration effect size calculator [24], examining the differences that comparator groups or recruitment settings may have on outcomes. Studies will be categorised by target disorder, with studies with multiple target disorders discussed in each category. Studies with multiple outcome measures per target disorder will be detailed in the narrative analysis. Meta-analyses will be conducted for each target disorder and comparator where data allows. The measure used in the meta-analysis was based on the following hierarchy: depression (PHQ-9, Hamilton rating scale for depression—HRSD, Beck depression inventory—BDI [20, 25, 26]), anxiety (GAD-7, State-trait anxiety inventory—STAI, Beck Anxiety Inventory and the Hospital Anxiety and Depression Scale [21, 27–29]), anger (NAS-PI, DAR, State-trait anger expression inventory—STAXI [23, 30, 31]), alcohol misuse (Audit, Timeline follow-back—TLFB [22, 32]) where multiple outcome measures have been used in studies. No general categories will be employed with studies being required to directly measure one of the four target disorders in order to warrant inclusion in the review. In order to control for upward bias, only target outcomes shall be analysed with secondary disorders identified through questionnaire battery not included.

### Methods for identification of studies

Electronic searches of CENTRAL, PsycInfo, MEDLINE, CINAHL, The Cochrane Register of Clinical Trials, EMBASE and ASSIA will be undertaken using free text, MESH headings and Boolean operators, with the search adjusted to fit each database. A proposed search strategy for the MEDLINE database is attached (Additional file 2).

Forward and backward citations will be explored by examining the reference lists of identified studies. A grey literature search will also be undertaken examining conference proceedings, grey literature databases and relevant websites. Subject authors will be contacted to identify missing data and any additional studies that meet the inclusion criteria. There will be no country exclusions for studies; however, only studies available in English will be included in the review.

The search process will begin with title and abstract examination by LO, with full papers then obtained and compared against the inclusion/exclusion criteria. A subset of the papers (10%) will also be reviewed by an independent reviewer not connected to the study in order to check reliability using a Kappa statistic [33]. Any discrepancies about study inclusion will be resolved through discussion in consensus meetings.

A PRISMA flow chart will be produced. The initial database searches will take place between June and July 2016. The next steps of the research process will depend on the number of studies identified. Any study published in English up to the date that the searches commence will be included in the review.

### Data extraction and analysis

A data extraction form will be produced and trialled. Missing data will be requested from authors. If it is not possible to obtain missing data, sensitivity analysis will be conducted to assess the impact of removing this study from the data analysis on any statistical synthesis of data. Data extraction will be undertaken by LO again checked by an independent reviewer to test for reliability. Any discrepancies will again be resolved through discussion at consensus meetings. Data to be extracted will cover the following areas: participant characteristics (number, age, gender, rank, regular or reservist etc.), service branch (Army, Navy, Airforce), intervention used (face-to-face high intensity therapy, self-help, guided help etc.), comparator group(s) (treatment as usual, alternative treatment, watchful waiting), outcome measure(s) used (PHQ-9, GAD-7, HADS, AUDIT, BDI, NAS-PI), study outcome (symptomatic change and any associated effect size) with discrepancies resolved through discussion. Follow-up data will be extracted where applicable. Standard mean difference between intervention group(s) and control post intervention, along with the respective 95% confidence intervals, will be calculated using the Campbell Collaboration effect size calculator using study outcome means, standard deviations and number of participants [24]. This will be done using between groups post intervention and follow-up (up to and including 6 months post intervention) means, standard deviations and number of participants, with missing data again requested from study authors. As the review only focuses on symptomatic change, dichotomous data will not be applicable.

### Risk of bias

Quality assessment will be conducted using the Cochrane Collaboration Tool [34]. The tool will allow the quality of identified studies to be discussed in a manner consistent with the Centre for Reviews and Dissemination guidelines [35]. Studies identified as poor quality will be dropped from the analysis in order to improve the reliability of findings.

Extracted data will be presented in a tabulated format. A risk of bias table will be compiled using RevMan [36].

### Data synthesis

Where a sufficient number of papers meeting inclusion criteria are identified, meta-analysis will be undertaken. Although meta-analysis can be conducted with as little as two studies, if a large amount of heterogeneity exists, concerns regarding the reliability of any statistical synthesis can arise. In addition to this, because a random effects model will be used, a small number of studies may lead to an error in the between studies variance estimation. The number of studies identified and the level of heterogeneity will therefore be taken into account when considering the suitability of the data for a meta-analysis. If a meta-analysis is deemed appropriate, effect sizes (standardised mean difference, Cohen’s *d*) and 95% confidence intervals will be calculated again using the Campbell Collaboration effect size calculator [24] with forest plots produced to visually represent findings. Effect size will be interpreted using Cohen’s classification (small, 0.20 to 0.49; medium, 0.5 to 0.79; large >0.80) [37].

In the event that only a small number of studies are identified with a large amount of heterogeneity present, a full narrative review will be undertaken using the ‘Narrative Synthesis in Systematic Reviews’ tool [38] which will allow for a textual approach to be adopted and applied, explaining the findings of the study data.

It is expected that a number of different interventions will be examined across studies. Therefore, a random effects model will be adopted, as advised by Borenstein [39]. Cochran’s Q statistics will be calculated to test for heterogeneity, with *I* ^2^ statistics calculated to quantify the degree of heterogeneity found. If at least 10 studies are identified (as per Cochrane handbook for systematic reviews of interventions advice [15]), funnel plots will be produced using RevMan [36] to check for publication bias with Eggers’ test of intercept used to determine if funnel plot asymmetry exists [40].

If heterogeneity exists, subgroup analysis will be conducted in order to determine whether this is due to methodological or participant factors. Moderators may include clinical presentation, branch of service, high/medium quality study, country of origin and early service leaver vs non-early service leaver, or methodological moderators such as recruitment source.

## Discussion

The effectiveness of existing interventions for depression, anxiety, alcohol misuse and anger in armed forces veterans and their families will be explored through the planned systematic review.

The two existing reviews [12, 13] in the area of armed forces veteran mental health have employed narrow searches. Therefore, the effectiveness of interventions addressing anger and alcohol misuse remains unknown. The proposed review will employ a robust method following PRISMA, Cochrane and CRD guidelines and a broad range of search terms used to establish a comprehensive picture of the research in the area to date.

Most research addressing the mental health of armed forces veterans has been conducted in the USA. This review will attempt to adopt a more inclusive approach by using the British definition of the term armed forces veteran, which may lead to the inclusion of studies that may have been excluded when a narrower definition of veteran, such as that used in the USA, is applied.

Given the prevalence and significant impact of poor mental health in armed forces veterans, results of this review may be important in guiding future research, service development, clinical practice and health care policy such as the armed forces covenant that focuses on the mental health of armed forces veterans and their families [18].

## Additional files

Additional file 1: PRISMA-P Checklist. (DOC 82 kb)

Additional file 2: Ovid MEDLINE Search Strategy. (DOCX 16 kb)

## Abbreviations

CBT: Cognitive behavioural therapy
PRISMA-P: Preferred Reporting Items for Systematic Reviews and Meta-Analyses Protocol
PROSPERO: International Prospective Register of Systematic Reviews
PTSD: Post-traumatic stress disorder
