# Supplementary material for: Psychological interventions for the treatment of depression, anxiety, alcohol misuse or anger in armed forces veterans and their families: systematic review and meta-analysis protocol
Source: Syst Rev. 2017 Jun 15;6:112. doi: 10.1186/s13643-017-0513-8 (PMC5472945; doi:10.1186/s13643-017-0513-8)
Supplement: Supplementary file 2 — Ovid MEDLINE Search Strategy. (DOCX 16 kb) [file 13643_2017_513_MOESM2_ESM.docx]

Search Strategy

1. Veteran*.ti,ab.
2. Retired.ti,ab.
3. Ex-serving.ti,ab.
4. Discharged.ti,ab.
5. Early service leaver.ti,ab.
6. Military.ti,ab.
7. Nav*.ti,ab.
8. Army.ti,ab.
9. Air Force.ti,ab.
10. Armed Forces.ti,ab.
11. Reservist.ti,ab.
12. Military Personnel/
13. Military Family/
14. Family Member*.ti,ab.
15. Dependant*.ti,ab.
16. Spous*.ti,ab.
17. Spouses/
18. Wife.ti,ab. OR Wives.ti,ab.
19. Partner$.ti,ab.
20. Parent$.ti,ab.
21. Mother$.ti,ab.
22. Father$.ti,ab.
23. Child*.ti,ab.
24. Caregiv*.ti,ab.
25. Caregivers/
26. (1 OR 2 OR 3 OR 4 OR 5 OR 6 OR 7 OR 8 OR 9 OR 10 OR 11 OR 12 OR 13)
27. (14 OR 15 OR 16 OR 17 OR 18 OR 19 OR 20 OR 21 OR 22 OR 23 OR 24 OR 25)
28. (26 AND 27)
29. Depress*.ti,ab.
30. Depression/
31. exp Depressive Disorder/
32. Melancholi$.ti,ab. OR Dysphori$.ti,ab. OR dysthymi$.ti,ab.
33. Low mood.ti,ab.
34. Anxiet*.ti,ab.
35. Anxiety/
36. exp Anxiety Disorders/
37. Stress.ti,ab. OR Worry.ti,ab.
38. Anxious.ti,ab.
39. Anger.ti,ab.
40. Anger/
41. Aggress*.ti,ab.
42. Aggression/
43. Violen*.ti,ab.
44. Violence/
45. Hostility.ti,ab.
46. Hostility/
47. Alcohol.ti,ab.
48. Alcohol misuse.ti,ab.
49. Problem Drinking.ti,ab.
50. Alcohol abuse.ti,ab.
51. Alcohol dependence.ti,ab.
52. Alcohol Drinking/
53. (29 OR 30 OR 31 OR 32 OR 33 OR 34 OR 35 OR 36 OR 37 OR 38 OR 39 OR 40 OR 41 OR 42 OR 43 OR 44 OR 45 OR 46 OR 47 OR 48 OR 49 OR 50 OR 51 OR 52)
54. (26 AND 53)
55. (28 AND 53)
56. CBT.ti,ab.
57. exp Counseling/
58. cognitive restructuring.ti,ab.
59. Cognitive reframing.ti,ab.
60. Behavio$ activation.ti,ab.
61. Activity Scheduling.ti,ab.
62. Problem Solving.ti,ab.
63. cCBT.ti,ab. OR iCBT.ti,ab.
64. Psychodynamic.ti,ab.
65. IPT.ti,ab.
66. Interpersonal Psychotherapy.ti,ab.
67. Behav*.ti,ab.
68. Cognitive Therapy/
69. Selfhelp.ti,ab. OR Self Help.ti,ab.
70. Selfmanag$.ti,ab. OR Self manag$.ti,ab.
71. Selfadminister$.ti,ab. OR Self administer$.ti,ab.
72. Psychosocial.ti,ab.
73. Psychotherapy.ti,ab.
74. Psychoeducation.ti,ab.
75. Guided help.ti,ab.
76. Cognitive.ti,ab.
77. Counsel*.ti,ab.
78. Psychiatric Rehabilitation/
79. Psychotherapy/
80. Behavior Therapy/
81. Psychotherapy, Brief/
82. Psychology, Military/
83. (56 OR 57 OR 58 OR 59 OR 60 OR 61 OR 62 OR 63 OR 64 OR 65 OR 66 OR 67 OR 68 OR 69 OR 70 OR 71 OR 72 OR 73 OR 74 OR 75 OR 76 OR 77 OR 78 OR 79 OR 80 OR 81 OR 82)
84. (54 AND 83)
85. (55 AND 83)
